# ELDER FAMILY FINANCIAL EXPLOITATION: EXPERIENCES WITH SOCIAL SERVICES

**DOI:** 10.1093/geroni/igz038.3352

**Published:** 2019-11-08

**Authors:** Tina R Kilaberia, Emma Fuhrman, Marlene Stum, Iris Freeman

**Affiliations:** 1 University of Minnesota - Twin Cities, Minneapolis, Minnesota, United States; 2 Minnesota State University, Mankato, Mankato, Minnesota, United States; 3 University of Minnesota, St. Paul, Minnesota, United States; 4 Mitchell Hamline School of Law, St. Paul, Minnesota, United States

## Abstract

Elder family financial exploitation (EFFE) has attracted the attention of scholars and professionals across disciplines. This qualitative study examines the experiences of help seeking by non-perpetrator family members with a focus on the role of social services. 15 in-depth interviews were examined in which social services were mentioned as being involved. Findings provide insight into the role and involvement of social services, whether wishes expressed by victims to participants made a difference in help-seeking, and gaps experienced. Participants described social services professionals as those who (1) received reports of exploitation; (2) provided education and served as a liaison with families; (3) conducted assessments, including cognitive assessment of elders; and (4) acted as connectors to other systems. In some cases, when elders were assertive about their wishes, they had results such as reporting exploitation or transferring power of attorney to non-perpetrator family members. In other cases, elders were prevented from taking such action because of undue influence by perpetrators, disregard of their wishes, due to being uninformed, or opposing helpful family members. Participants explained experienced gaps in two ways: by attributing responsibility to social services in terms of failure to believe victims, do meaningful cognitive assessments, and navigate family dynamics. On the other hand, participants were not able to clearly ascribe responsibility, and questioned whose fault it was, suggesting opportunities for improved systems functioning. Recommendations for improving the role of social services in addressing the help-seeking needs of concerned family members coping with EFFE follow.

